# Wettability, Tribology, Degradation, and Topography of Laser-Textured Surfaces of Biopolymers

**DOI:** 10.3390/mi16091009

**Published:** 2025-08-31

**Authors:** Ciprian-Dumitru Ciofu, Petronela-Daniela Rusu (Ostahie), Marcin Adamiak, Oktawian Bialas, Catalin Tampu, Panagiotis Kyratsis, Anastasios Tzotzis, Simona-Nicoleta Mazurchevici, Alexandra Nedelcu, Zhengyi Jiang, Daniel Mindru, Dumitru Nedelcu

**Affiliations:** 1 Faculty of Mechanical Engineering, “Gheorghe Asachi” Technical University of Iasi, Street Prof. Dr. Doc. Dimitrie Mangeron, Nr. 43, 700050 Iasi, Romania; ciprian-dumitru.ciofu@academic.tuiasi.ro; 2 Faculty of Machine Manufacturing and Industrial Management, “Gheorghe Asachi” Technical University of Iasi, Street Prof. Dr. Doc. Dimitrie Mangeron, Nr. 59A, 700050 Iasi, Romaniasimona-nicoleta.mazurchevici@academic.tuiasi.ro (S.-N.M.); teodor-daniel.mindru@academic.tuiasi.ro (D.M.); 3 Faculty of Mechanical Engineering, Silesian University of Technology, Akademicka 2A, 44-100 Gliwice, Poland; marcin.adamiak@polsl.pl (M.A.); oktawian.bialas@polsl.pl (O.B.); 4 Faculty of Engineering, “Vasile Alecsandri” University of Bacau, Calea Mărășești 157, 600115 Bacău, Romania; catalin.tampu.ub@gmail.com; 5 Active Urban Planning Zone (ZEP) Kozani, University of Western Macedonia, 50 150 Kozani, Greece; pkyratsis@uowm.gr (P.K.); a.tzotzis@uowm.gr (A.T.); 6 Faculty of Dental Medicine, University of Medicine and Pharmacy “Grigore T. Popa” of Iasi, Strada Universității 16, 700115 Iași, Romania; alexnedelcu_10_2000@yahoo.ro; 7 University of Wollongong, Northfields Ave, Wollongong, NSW 2500, Australia; jiang@uow.edu.au; 8 Technical Sciences Academy of Romania, Blvd. Dacia 26, 030167 Bucharest, Romania; 9 Academy of Romanian Scientists, Ilfov Street 3, Sector 5, 050044 Bucharest, Romania

**Keywords:** Arboblend V2 Nature, surface texturing, WCA, friction coefficient, wear track, topography

## Abstract

Surface texturing involves creating micro-channels, micro-dimples, micro-grooving, and other surface modifications. To do this, laser and micromachining are employed on the substrate surface in addition to other methods. The surface characteristics of the Arboblend V2 Nature biodegradable polymers with laser texturing, hexagonal and square patterns, and four and six passes are shown in this study. Regardless of the texture type, Arboblend V2 Nature’s hydrophilic surface (a contact angle of less than 90°) was demonstrated by the results of the wettability test. The underlying material’s wear behavior changed as a result of the LST surface modification. The COF values increased only after six passes with both textures. On the topographical side, Arboblend V2 Nature (square and hexagonal) shows a consistent X-axis expansion in the hexagonal geometry and a considerable amount of variability in the square geometry, especially at six passes, where the Y-axis (higher depths) is more compressed. According to the results, since textured surfaces are practicable, non-biodegradable polymers from a variety of industries can be substituted.

## 1. Introduction

The technique known as surface texturing can be used to achieve the desired pattern on the surface. The material’s mechanical and tribological features include improved fatigue strength, corrosion resistance, wear resistance, antibiofouling hydrophobicity, and load-carrying capacity, to name a few. Metals [1,2], polymers [3], and ceramics [4,5] are among the technical materials that have been successfully treated by laser surface texturing (LST). This approach has been successful in several technological domains, such as coating, tribology, and biomedicine. To improve the material’s tribological behavior, LST has created a variety of textures and patterns. The procedure also raises the material’s coefficient of friction and wear resistance. A handful of the patterns comprise micro-grooves and dimples with variable sizes and forms [6,7,8].

Biomedical applications make extensive use of biopolymers, which are both synthetic and natural polymers [1,9]. Their exceptional mechanical qualities and biocompatibility, which in some circumstances can be equivalent to human tissues, are the cause of this. Polyethylene (UHMWPE) materials with a carbon coating were subjected to three distinct laser wavelengths: 1.064 µm, 532 nm, and 355 nm. Lasers with wavelengths of 355 nm and 532 nm are relatively appropriate for enhancing the surface conditions of UHMWPE, such as surface roughness and wettability.

Although the CO_2_ laser texturing of poly (l-lactide) presents an opportunity to modify the material surface’s physical and structural characteristics to meet the needs of the cells, it also significantly alters the treated polymer surface’s mechanical characteristics [10].

Using lasers with wavelengths of 1.064 µm, 355 nm, and 532 nm, the effects of lasers on the wettability, roughness, and hardness of polypropylene material were examined.

The authors proposed roughening the polypropylene surface with laser wavelengths [11]. According to the literature, to improve the bone bonding on the implant surface, Ra values greater than 1 µm are at least necessary.

Mirzadeh et al., [12] improved the hydrophilicity and biocompatibility of ethylene–propylene rubber N-vinylpyrrolidone (NVP) and 2-hydroxyethyl methacrylate (HEMA) by subjecting the polymer to a pulsed CO_2_ laser at varying laser intensities. Koufaki et al., [13] investigated the possibility of cell adhesion on high-rough polymeric surfaces with a gradient roughness ratio and wettability produced by laser micro/nano-textured Si surfaces. They found that both cell types adhered more successfully to microstructured surfaces connected to unstructured surfaces.

Furthermore, PC12 cells were shown to attach well to the patterned surface [14]. After four days, when the cells multiplied into a merging patch inside the channels, direct laser writing on the biodegradable polymer to create micro-channels for the attachment of C_2_C_12_ myoblast cells in the micro-channel demonstrates a high degree of alignment [14]. The surface properties and features of nylon 6.6 treated with a CO_2_ laser were described in detail in the paper by Waugh et al. [15]. They proposed that laser-textured surfaces enhance nylon 6.6’s biomimetic properties in regard to the osteoblast cell response.

Only a small number of polymeric biomaterials are currently being utilized in clinical practice, and many have been investigated for tissue engineering [16,17,18].

When it comes to bodily fluids, Ultra-High-Molecular-Weight Polyethylene (UHMWPE) is non-toxic, chemically stable, and highly resistant to wear and impact. The roughness and wettability are increased by laser treatments. Regardless of the laser wavelength, Lorusso et al. [19] discovered that the WCA decreased as the number of pulses increased. In their study, Riveiro et al. [20] modified UHMWPE using a variety of laser wavelengths (1064, 532, and 355 nm); to lessen the material’s high transparency at these laser wavelengths, a carbon coating was applied to the UHMWPE samples’ surface. For many years, this substance has been utilized as a bearing material in joint replacement implants. In biological applications, such as hip or patella prostheses, it is frequently utilized.

- An amorphous thermoplastic polymer with a high transparency to visible light is called polycarbonate (PC) [21]. The first people to conduct LST on PC, specifically on PC/PMMA blends, were Viville et al. The purpose of this study was to assess the chemical and physical changes made to the surfaces after the laser treatment. For this purpose, a KrF excimer laser was used to emit a UV laser beam with a pulse length of 30 ns. UV light was shown to be capable of producing chemical changes and micropatterns on PC/PMMA mix surfaces [21,22]. This substance is suitable for use in biomedical applications. PC has been employed as a biomaterial in a variety of applications, from heart surgery to renal dialysis.
- By using a UV laser, polytetrafluoroethylene (PTFE) was altered. The procedure was carried out by Ahad et al. in a nitrogen-rich setting. A precisely regulated surface patterning was the outcome of the treatment [23].
- A CO_2_ laser was also used to texture nylon 6.6 to improve its biocompatibility. The biological investigations showed that the nylon’s cell survival was impacted by the surface alteration when employing this laser wavelength. Surfaces treated with CO_2_ lasers showed enhanced cell proliferation [24].

Textured surfaces can be successfully used in various medical devices. Thus, Yasaka K. et al. [25] studied the effects of textured surfaces on plastic materials in order to reduce friction between the body tissues and components used. The results indicated that textured surfaces exhibited lower friction compared with normal surfaces for soft plastic materials. Ikeuchi K. et al. [26] studied the tribological properties of polyurethane grafted (DMAA) in water. A DMAA-coated medical device may allow for precise functioning, painless insertion, and, last but not least, tissue protection. According to Shivakoti et al. [8], LST has a lot of promise for use in biological applications these days, with a particular emphasis on polymers. Mirzadeh et al. [12] increased the hydrophilicity and biocompatibility of ethylene–propylene rubber N-vinylpyrrolidone (NVP) and 2-hydroxyethyl methacrylate (HEMA) by grafting to the polymer with a pulsed CO_2_ laser at different laser intensities. Alveolar macrophages grown on untreated films exhibit better adherence to cells with good propagation and flattening, whereas supports attached to the treated EPR exhibited circular shapes with limited cytoplasmic spreading and ruffling. According to a study by Koufaki et al. [13] on the potential for cell adhesion on high-rough surfaces with gradient roughness ratios and wettability generated by laser micro/nano-textured Si surfaces, both cell types adhered more successfully to microstructured surfaces connected to unstructured surfaces. Furthermore, it was shown that PC12 cells adhered to the patterned surface with remarkable efficiency. According to Evangelista et al. [27], current equipment usually has to be updated without compromising its key capabilities or design aspects in order to maximize the tribological qualities of medical devices. The functioning of a medical device is always affected by surface contacts, which are associated with tribology studies. Compared to devices with no relative motion between their various components, biomedical applications involving continuous mechanical motion—such as prosthetic implants, artificial joints, and moving mechanisms in drug delivery devices—generally have higher failure rates because of wear and friction [28,29,30]. The possible use of laser texturing to create surface-infused nanogold particles with PEEK was examined by Biala et al. [31]. The primary characteristics of the design for biomedical applications were biomimetic patterns and parameter optimization.

The biodegradable biopolymer used (Arboblend V2 Nature) was patented by a group of researchers from the Fraunhofer Institute for Chemical Technology (ICT) in Pfinztal, Germany, in association with the business Tecnaro GmbH. A vast array of material kinds and property profiles are combined in ARBOBLEND^®^. The available materials are as much as 100% bio-based. Polyhydroxyalkanoates (PHAs), polycaprolactone (PCL), polyester (especially bio-PET), starch, polylactic acid (PLA), bio-polyolefins (bio-PEs), bio-polyamides (bio-PAs), lignin, natural resins, natural waxes, natural oils, natural fatty acids, cellulose, organic additives, and natural reinforcing fibers are among the biopolymers found in ARBOBLEND^®^ materials, depending on the formula. Depending on the use, ARBOBLEND^®^ materials are made to be either resistant or biodegradable. One more advantage of biodegradability is that it can be used for mulch films, plant assistance, urns, and many more applications [32].

There are a number of benefits and drawbacks to using the biodegradable biopolymer in this research for laser texturing. As a bio-composite material made from renewable resources, its environmentally friendly makeup satisfies the growing need for sustainable production methods. In addition to functionalizing material surfaces, the microtexturing technique reduces waste, making it a sustainable option that complies with pro-environmental regulations. Through the use of laser technology, the material may be precisely and intricately texturized, allowing for unique surface finishes, functional surface enhancements, and personalized designs that are challenging to accomplish with traditional metals or polymers. It can endure the localized heating effects of laser texturing without suffering considerable distortion because it also demonstrates strong thermal stability during processing. Its machinability also lessens equipment wear and makes laser processing operations run more smoothly.

Characterizing the obtained sample and texturing the surfaces (four and six passes) of the parts obtained using injection molding technology (Arboblend V2 Nature biodegradable materials), with hexagonal and square shapes, is the goal of this study. The novel features of this work are the improved surface characteristics and texturing of the biodegradable Arboblend V2 Nature materials. The mechanical, thermal, and structural properties of the materials mentioned in the studies were investigated by several researchers [33,34,35,36].

## 2. Materials and Methods

### 2.1. Samples Preparation

Arboblend V2 Nature is a biopolymer patented by Tecnaro GmbH in collaboration with researchers at the Fraunhofer Institute for Chemical Technology, Pfinztal, Germany. The initial samples were made by injection molding using the SZ-600 H injection machine (Shen Zhou, Zhangjiagang, China). The TERGAMIN-30 grinding–polishing machine (Struers, Willich, Germany) was utilized for mechanical finishing before laser micromachining. During a time of 4 min, the samples were mechanically polished and successively polished using paper grain sizes of 500, 800, and 1200 grid/mm^2^. The 9, 3, and 1 μm samples were then mechanically polished using polishing wheels.

### 2.2. Laser Surface Texturing (LST)

The surface texturing process was conducted using an A-355 picosecond laser system (Oxford Lasers Ltd., Didcot, UK). The processing parameters are presented in Table 1. The laser pattern, often referred to as the filling strategy, was constructed using the Cimita software (Oxford Lasers, Didcot, UK) built into the micromachining apparatus.

The codes used for textured surfaces are as follows: 4x_H = hexagonal pattern, 4 laser passes; 4x_S = square pattern, 4 laser passes; 6x_H = hexagonal pattern, 6 laser passes; and 6x_S = square pattern, 6 laser passes.

Table 2 presents the tests carried out on laser surface texturing.

## 3. Results and Discussions

### 3.1. Microscopic Observation

**Figure 1:**
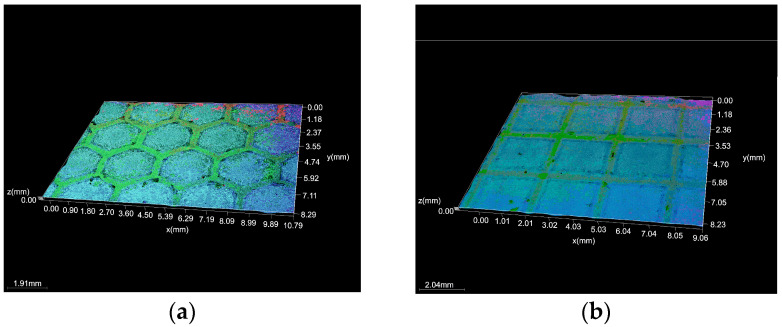
Microscopic observation of biodegradable biopolymer after laser texturing process: (**a**) 4x_H x300 and (**b**) 4x_S x300.

**Figure 2:**
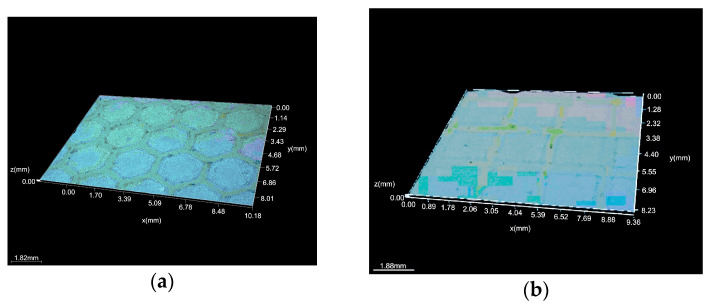
Microscopic observation of biodegradable biopolymer after laser texturing process: (**a**) 6x_H x300 and (**b**) 6x_S x300.

Figure 1 and Figure 2 display the samples’ surface morphology following the laser texturing procedure. Honeycomb (group H) and cross-like micro-groove (group S) patterns, which are indicative of photothermal ablation, were seen in all analyzed samples, irrespective of the kind of base material groupings. In this way, the wavy profile that makes up the texture was shown via transverse and parallel cuts. The dimples’ diameter was perfectly uniform and complemented the design. According to the microscopic examination, the grooves exhibit common laser-induced surface patterns that result from the laser beam’s contact with the sample surface. It has been determined that the textured surfaces produced by laser ablation are continuous grooves with a regular, explosively vaporized bottom.

### 3.2. Wettability Test

**Figure 3:**
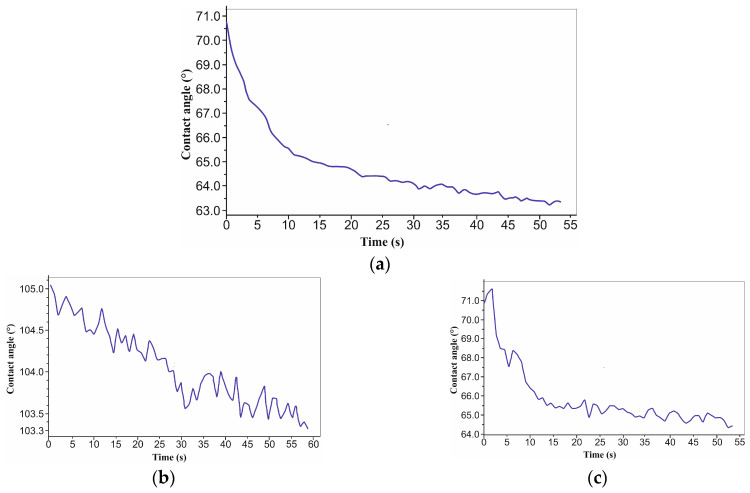
Contact angle diagram 4x: (**a**) initial material; (**b**) S; and (**c**) H.

**Figure 4:**
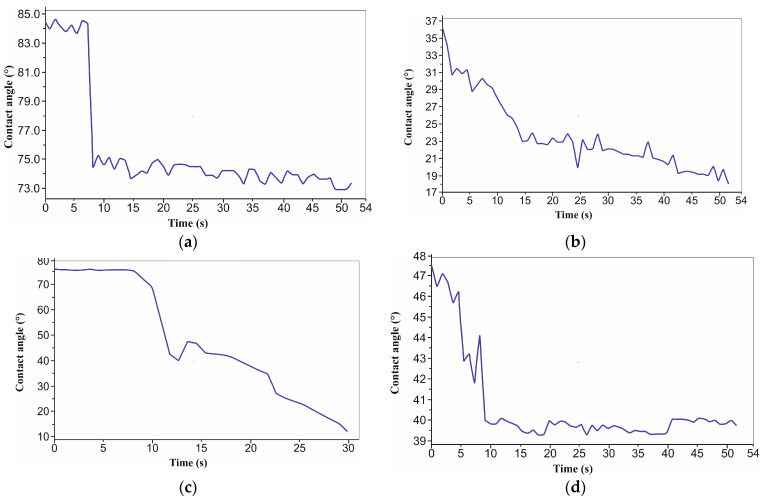
Contact angle diagram 6x: (**a**,**b**) S and (**c**,**d**) H.

The hydrophilic nature of the surface (contact angle less than 90°) was indicated by the samples’ initial condition—a mean distilled water wetting angle of 63 ± 1° (Figure 3a). It was discovered that the laser texturing surface modification changed the samples’ surface chemical makeup. Similar surface characteristics were noted for the experiment with the 4x and H texture pattern sample group (Figure 3c), and the average water contact angle was 62 ± 2°. Conversely, a group increase in the water contact angle was noted for the experiment involving the 4x and S texture pattern samples (Figure 3b). A mean water contact angle of 98 ± 4° indicates that the samples’ surfaces are hydrophobic. In comparison to the samples in the initial state, the water contact angle decreased for the experiment with the 6x and H texture pattern sample group (Figure 4c,d), with a mean value of 30 ± 8°. The standard deviation, which is 80% of the average value of all measures, needs special attention though. Absolute hydrophilic properties were also noted during measurement 1. This implied a heterogeneous surface for the sample. Hydrophilic characteristics were noted for the 6x and S texture pattern sample group experiment (Figure 4a,b); the average contact angle was 68 ± 5° (Table 3).

### 3.3. Wear Test

**Figure 5:**
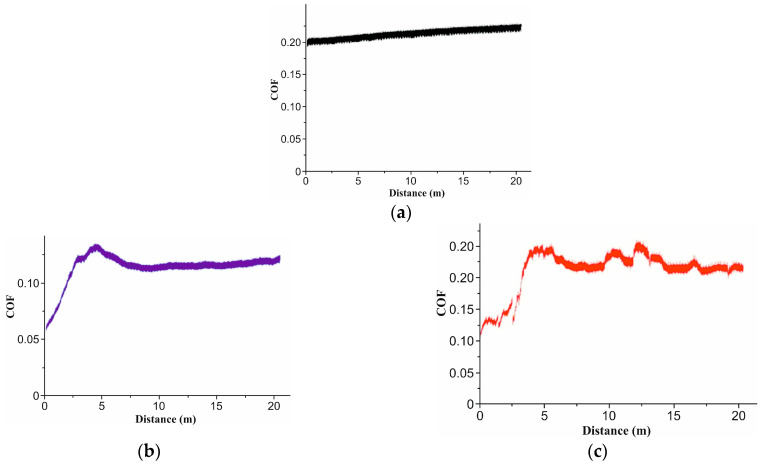

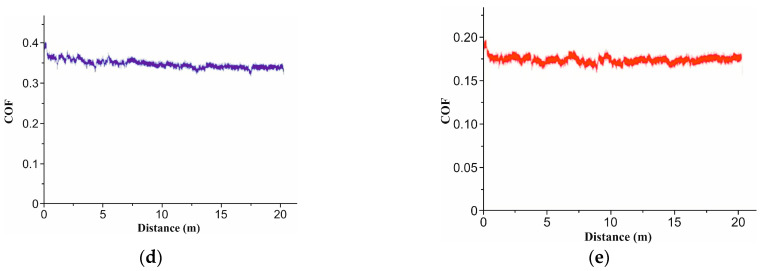
COF values: (**a**) initial material; (**b**) 4x_H; (**c**) 4x_S; (**d**) 6x_H; and (**e**) 6x_S.

**Figure 6:**
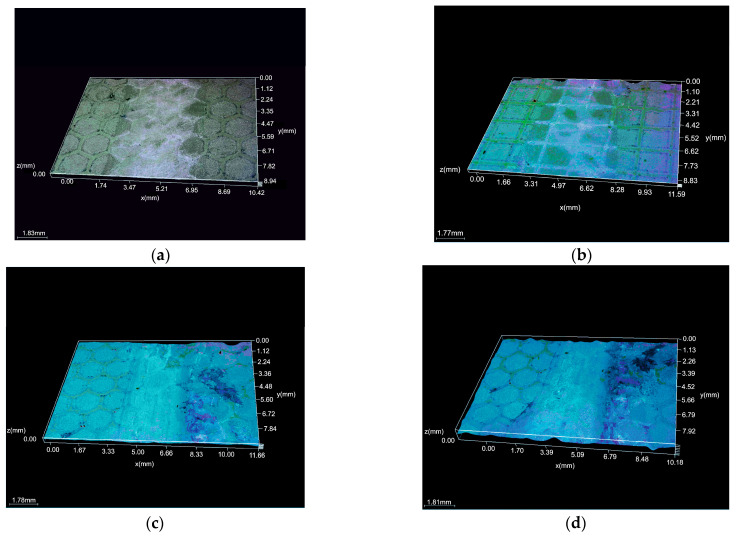
Wear track morphology: (**a**) 4x_H x200; (**b**) 4x_S x200; (**c**) 6x_H x200; and (**d**) 6x_S x200.

Figure 5 compares the evolution of the COF for the pristine polymer (a) and for laser-textured surfaces with hexagonal (H) and squared (S) morphologies at two scan densities (4× and 6×). All textured samples show a short running in stage, followed by stabilization, but the plateau level depends on both the morphology and scan density. At the lower scan density (4×), the hexagonal texture (b) attains the lowest and relatively stable COF, stabilizing near ~0.10–0.12 after the first few meters. At the same 4× density, the squared texture (c) stabilizes at a higher and more fluctuating level (~0.20–0.22). At the higher scan density (6×), the trends invert in magnitude: the hexagonal texture (d) exhibits the highest COF (~0.32–0.34) with a slight downward trend, whereas the squared texture (e) maintains a lower and stable plateau (~0.17–0.19). We interpret this crossover as an interplay between the geometry and laser-induced surface modification. At 4×, the rounded hexagonal features reduce the real contact area and effectively trap debris, promoting a continuous transfer film and thus a low COF. At 6×, the stronger laser exposure likely increases near the surface crosslinking/oxidation and roughness/edge definition of the hexagonal features, leading to asperity microfractures and unstable film renewal, which elevates the COF; the squared texture at 6×, despite sharper edges, appears to form a more persistent transfer film and shows a lower, steadier COF. The textured pattern was not removed throughout the wear test under the suggested conditions for all samples following the laser texturing procedure, according to the microscopic examination (Figure 6).

It was found that wear debris produced during the wear process may become trapped inside the micro-groove structure for every sample group that was evaluated. As a result, there is less wear debris on the surface, which eventually improves the wear behavior. The enhanced surface roughness in comparison to the base material may be the reason for the higher COF values for samples that were recorded following the laser texturing procedure.

### 3.4. DSC Analysis

**Figure 7:**
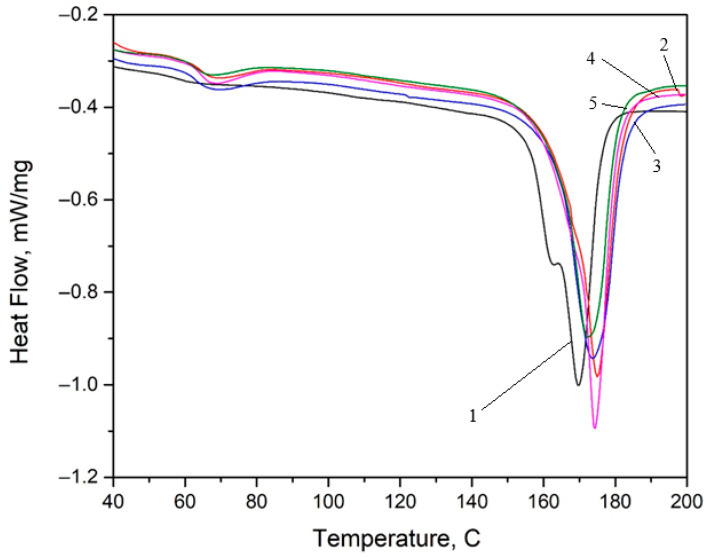
Heat flow variation with temperature during heating for all samples: 1—base material; 2—6x_S; 3—6x_H; 4—4X_S; and 5—4X_H.

The DSC thermograms recorded during heating are shown in Figure 7. During heating, two transformations were recorded on all analyzed samples: the glass transition in the temperature range of (60–70) °C and the melting in the temperature range of (150–180) °C. For the glass transition, the start temperature (T_sgt_), the midtemperature (T_midgt_), the inflection point (T_igt_), the end temperature of the transformation (T_fgt_), and the specific heat variation (ΔCp) were determined. For the melting, the start temperature (Ts), the temperature at which 50% of the melting took place (T50), the end temperature (Tf), and the amount of heat absorbed (ΔH) were identified (Table 4).

### 3.5. Degradation Test

**Figure 8:**
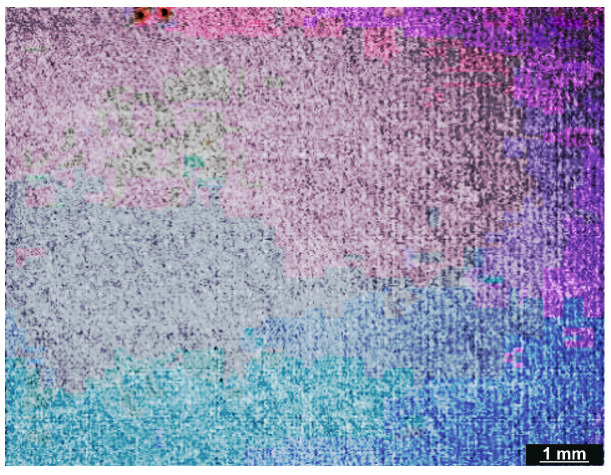
Microscopic observation of the initial surface after the degradation test.

**Figure 9:**
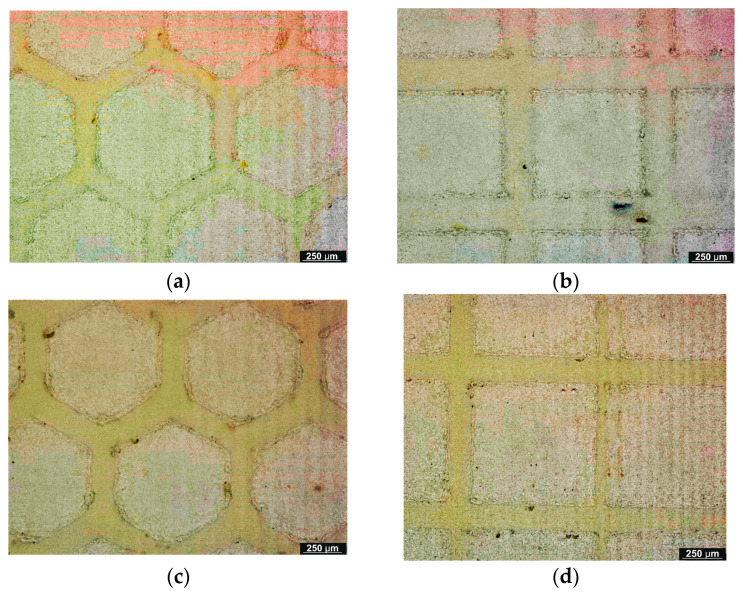
Microscopy of LST surfaces after degradation test: (**a**) 4x_H; (**b**) 4x_S; (**c**) 6x_H; and (**d**) 6x_S.

All examined samples showed an increase in weight, according to the weight measurements taken before and after the degradation test (Table 5). In general, if the polymer materials’ weight increases during a degradation test, it may be a sign of incomplete breakdown; if the degradation process is not finished, residues from partially broken-down polymer molecules may still be present and contribute to the weight increase. The microscopic analysis of each sample after the degradation test, however, revealed no degradation products (Figure 8 and Figure 9). When polymer-based materials interact with the testing environment (degradation conditions), they may experience chemical reactions that result in weight gain. Polymer chains may occasionally cross-link as a result of degradation processes, adding to the material’s weight.

### 3.6. Topography

#### 3.6.1. Topographic Analysis of Hexagonal Texture

##### Topographic Analysis of Surfaces with 4x_H

**Figure 10:**
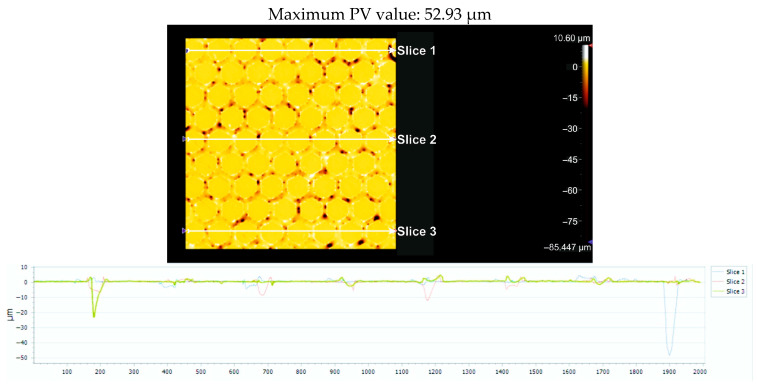
Maximum PV value from the analysis of the three sections: 4x_H.

For each of the three sections under examination, Figure 10 displays the greatest distance (PV_Peak and Valley) between the lowest and highest peak (PV_Peak and Valley) for the Arboblend V2 Nature material with the hexagonal texture, which was achieved by four passes (4x). Table 6 displays the relevant statistical information.

**Figure 11:**
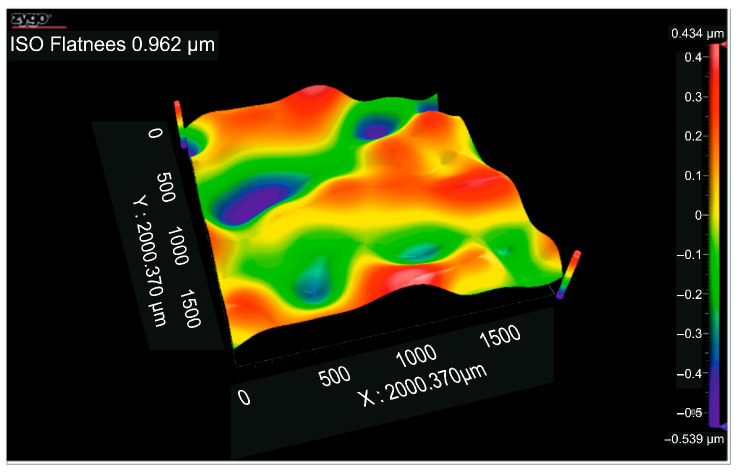
Flatness deviation after texturing: 4x_H.

**Figure 12:**
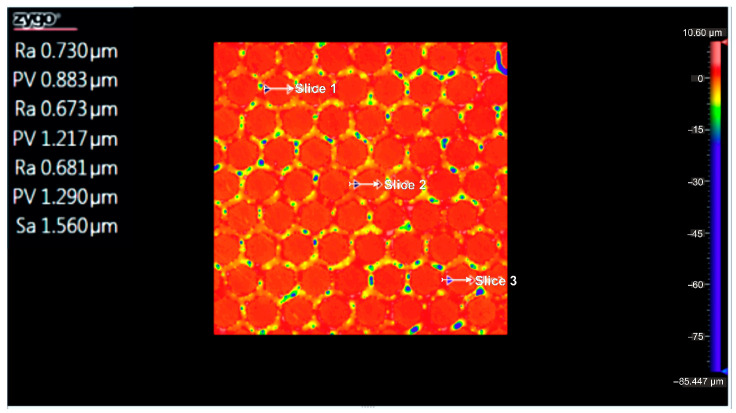
Roughness value Ra for three reference lines: 4x_H.

Figure 11 displays the flatness deviation following texturing, which, according to the ISO Flatness, is 0.962 µm. Figure 12 displays the roughness values R_a_ for the three selected reference lines, which solely correlate to the obtained hexagonal surfaces without accounting for the spacing between hexagons.

**Figure 13:**
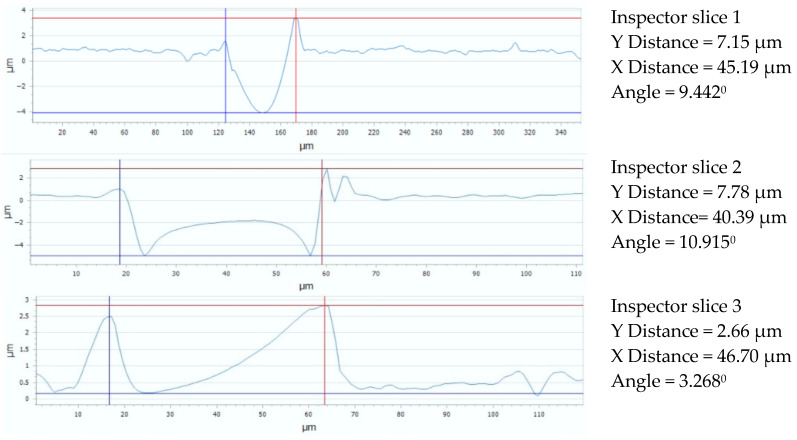
Gap value for three reference lines: 4x_H.

For the Arboblend V2 Nature material with a hexagonal lattice structure (like a honeycomb), Figure 13 displays three sets of data and graphs pertaining to the geometry and observed distances. A graph and details on the distances measured in the X and Y directions are included with each of the three sections. The following findings are derived from a comparative analysis of the data presented in Figure 13: The greatest distance fluctuations are observed along the OX axis, with the highest and lowest values in Section 3 (slice 3, 46.70 μm) and Section 2 (slice 2, 40.39 μm), respectively. With differences of up to 6 μm between sections, this shows significant variability in the hexagonal structure in the horizontal direction. The lowest but still significant variations are found between the three sections along the OY (depth) axis. In contrast to the other two sections (7.15 and 7.78 μm), Section 3 (slice 3) exhibits significantly greater compression (subsidence) (2.66 μm). This points to a potential region of higher density in the network and implies a lesser but still considerable fluctuation along the vertical axis. A heterogeneous structure with areas of stretching and compression inside the lattice is suggested by the size differences and variations in the geometric profile; this could be significant for material investigation.

##### Topographic Analysis of Surfaces with 6x_H

**Figure 14:**
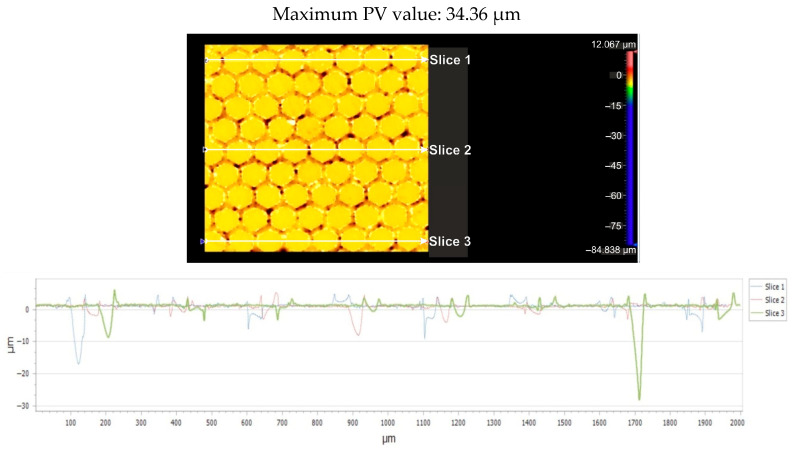
Maximum PV value from the analysis of the three sections: 6x_H.

Figure 14 displays the greatest distance (PV_Peak to Valley) between the lowest and highest peak (PV_Peak to Valley) for the three sections under consideration in the instance of the Arboblend V2 Nature material with the hexagonal texture, which was obtained by six passes (6x). Table 7 presents the statistical data that correlate to this distance.

**Figure 15:**
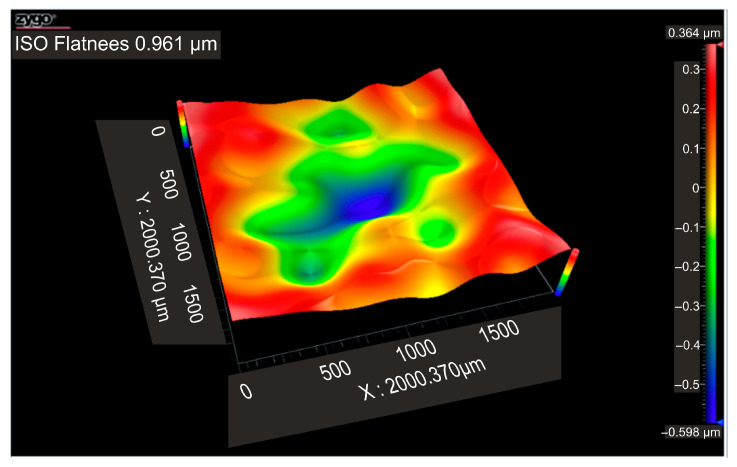
Flatness deviation after texturing: 6x_H.

**Figure 16:**
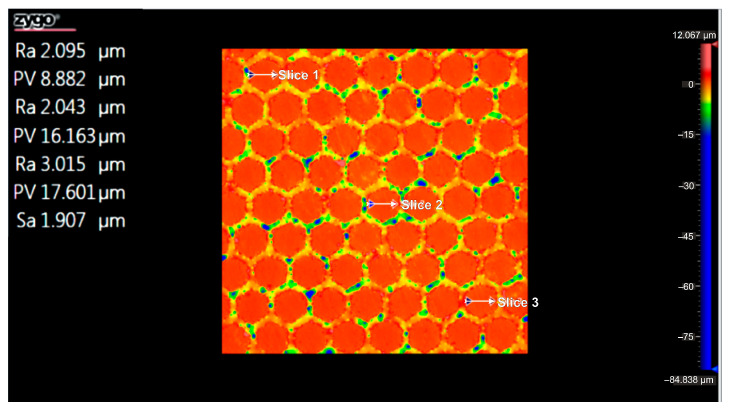
Roughness value Ra for three reference lines: 6x_H.

Figure 15 shows the flatness deviation after texturing, which, according to the ISO Flatness, is 0.961 µm. The roughness values Ra for the three selected reference lines are displayed in Figure 16; these values solely correspond to the produced hexagonal surfaces and do not account for the spacing between hexagons.

**Figure 17:**
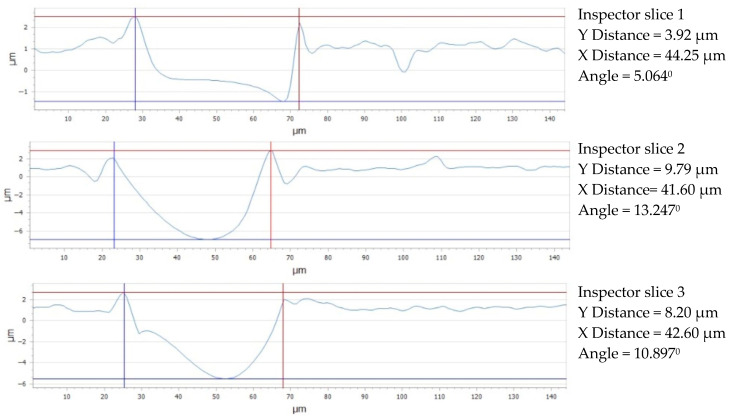
Gap value for three reference lines: 6x_H.

The following conclusions can be made by examining the data shown in Figure 17: Section 2 (slice 2) has the lowest distance (41.60 μm), while Section 1 (slice 1, along the OX axis) has the largest X-axis openness (44.25 μm). The fact that Section 3 (slice 3) falls between these two values indicates that the three sections have a comparatively homogeneous structure with just a minor X-axis variance. Section 2 has the largest distance (9.79 μm) between the lowest and the highest peak (PV) along the vertical axis; Section 3 lies between these two values, with a distance of 8.20 μm. Section 1 has the smallest distance along the Y-axis (3.92 μm), indicating a material thinning in this direction along the OY axis, in contrast to the X-axis, where the distances are closer; the Y-axis shows a greater fluctuation across the three parts of the Arboblend V2 Nature material processed with 6x.

The following conclusions can be made by comparing the two models, 4x_H and 6x_H: in the case of the X-axis, the distances are between 45.19 μm and 46.70 μm when processed with 4x and between 41.60 μm and 44.25 μm when processed with 6x. In the case of more passes (6x), there is a general tendency for decreasing distances on the X-axis. In the instance of the Y-axis, the distances range from 2.66 μm to 7.78 μm when machined with 4x and from 3.92 μm to 9.79 μm when machined with 6x. This could suggest a minor material thinning because of the heat produced by more passes. Unlike the X-axis, the Y-axis shows a tendency of rising distances as the number of passes grows from 4x to 6x, suggesting that the structure may extend on the vertical (depth) axis.

#### 3.6.2. Topographic Analysis of Square Texture

##### Topographic Analysis of Surfaces with 4x_S

**Figure 18:**
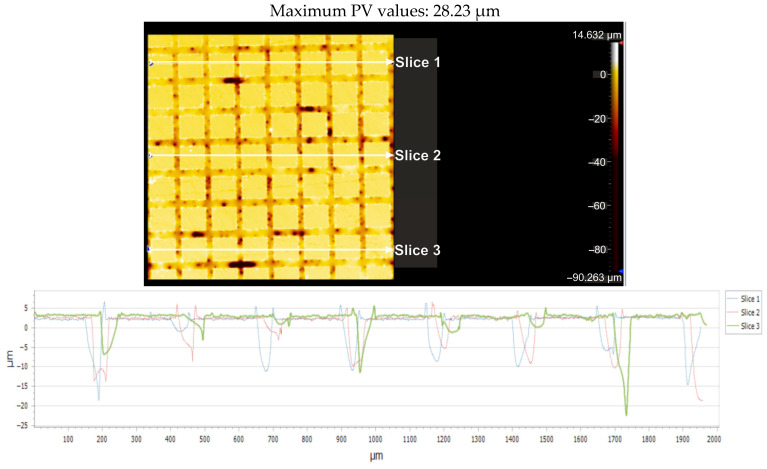
Maximum PV value from the analysis of the three sections: 4x_S.

For the Arboblend V2 Nature material with a square texture, which was produced by four passes, Figure 18 displays the greatest distance (PV_Peak to Valley) between the lowest and highest peak (PV_Peak to Valley) for each of the three parts that are being considered. Table 8 presents the pertinent statistical information.

**Figure 19:**
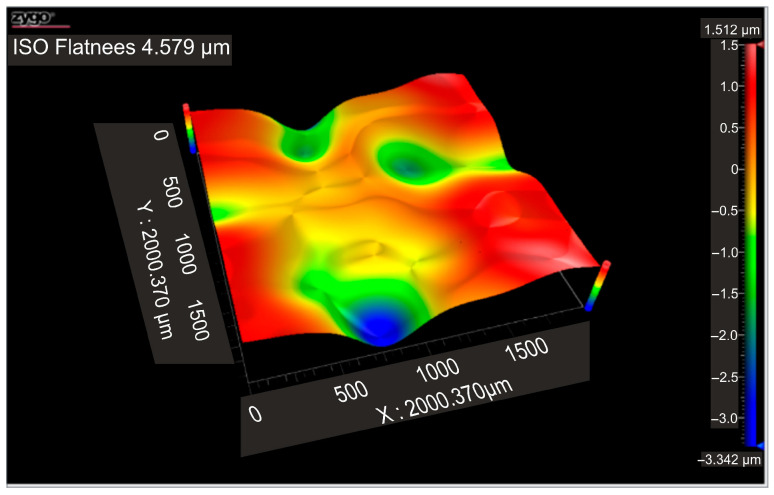
Flatness deviation after texturing: 4x_S.

**Figure 20:**
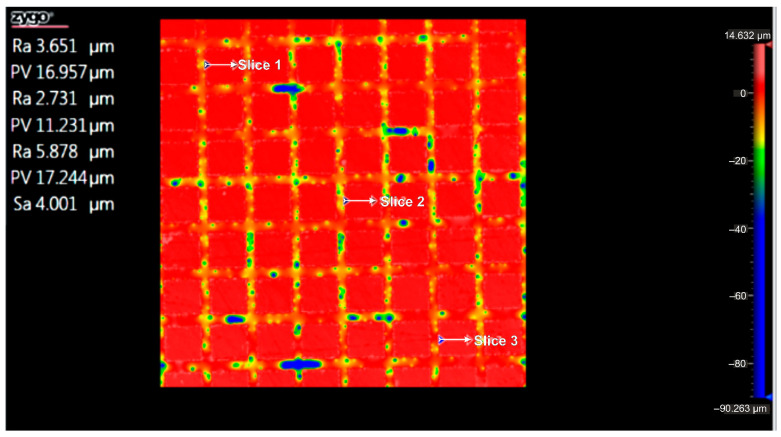
Roughness value Ra for three reference lines: 4x_S.

According to the ISO Flatness, the flatness deviation after texturing is 4.579 µm, as shown in Figure 19. Figure 20 shows the roughness values R_a_ for the three reference lines that were chosen. Without taking into account the distance between them, these values only relate to the square surfaces that were produced.

**Figure 21:**
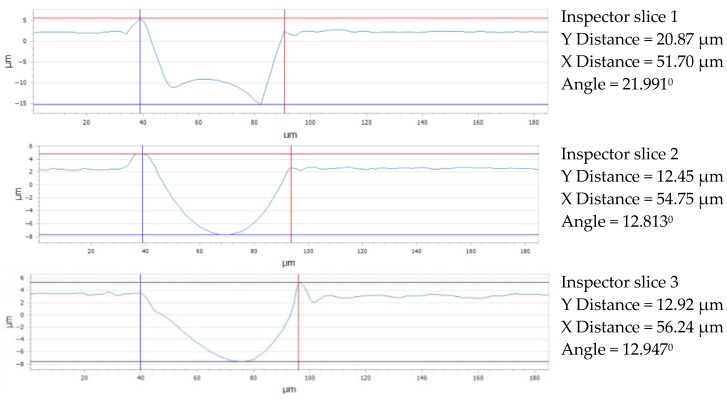
Gap value for three reference lines: 4x_S.

For the Arboblend V2 Nature material with a square lattice structure, Figure 21 displays three sets of data and graphs pertaining to the geometry and observed distances. A graph and details about the measured distances in the X and Y directions are included with each of the three sections. The following findings are drawn from a comparative examination of the data shown in Figure 21. The structure is relatively extended along the OX axis, as shown by the 51.70 μm aperture in Section 1 (slice 1), an even greater material thickening along the X-axis is suggested by the larger value of 54.75 μm in Section 2 (slice 2), and a very extended structure along the horizontal axis in this region is indicated by the largest aperture in Section 3 (slice 3), 56.24 μm. As we move from Section 1 to Section 3 (top to bottom), this distribution along the X-axis shows a trend of constant material thickening; along the Y-axis (depth), Section 1 has a large distance of 20.87 μm, indicating a significant material thickening along the vertical axis. Section 3 has a value very near to Section 2 at 12.92 μm, indicating a highly uniform structure along the Y-axis with the lowest material thinning, whereas Section 2 has a large drop in distance along the Y-axis at 12.45 μm, suggesting material thinning in this direction.

##### Topographic Analysis of Surfaces with 6x_S

**Figure 22:**
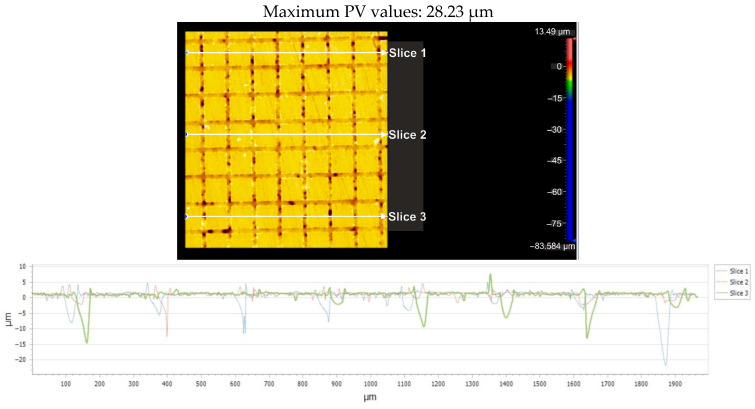
Maximum PV from three sections analysis: 6x_S.

**Figure 23:**
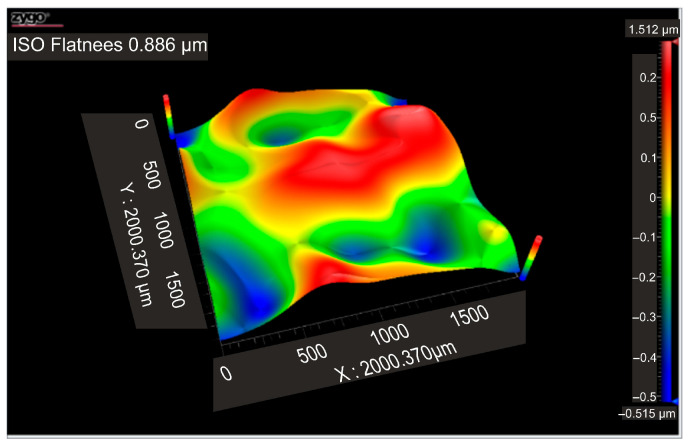
Flatness deviation after texturing: 6x_S.

**Figure 24:**
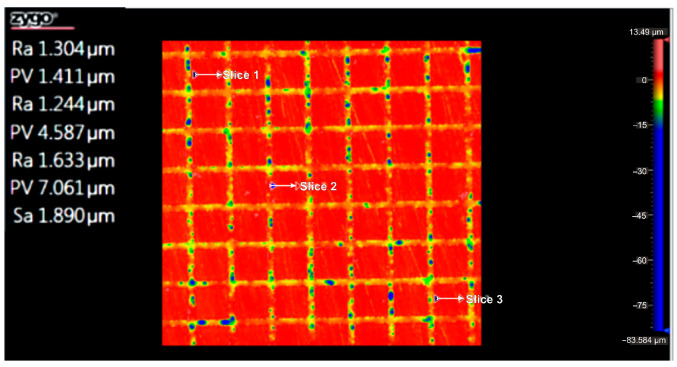
Roughness value Ra for three reference lines: 6x_S, on a geometric shape.

The maximum distance (PV_Peak to Valley) between the lowest and highest peak (PV_Peak to Valley) for each of the three sections under consideration is shown in Figure 22 for the Arboblend V2 Nature material with the square texture, which was obtained by six passes. The relevant statistical data are shown in Table 9. The Flatness deviation after texturing is shown as Figure 23. The Roughness value Ra for three reference lines is shown as Figure 24.

**Figure 25:**
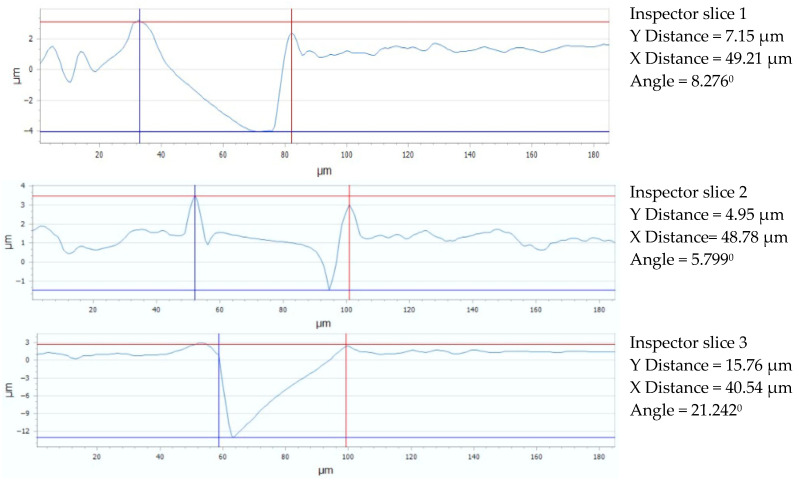
Gap values for three reference lines: 6x_S.

The following findings are drawn from a comparative examination of the data shown in Figure 25: a moderately extended structure on the horizontal axis is indicated by Section 1’s opening of 49.21 μm along the OX axis; a slight material thinning on this axis is indicated by Section 2’s (slice 1) slightly smaller value of 48.78 μm; and significant material thinning on the X-axis is suggested by Section 3’s (slice 3) smallest distance of 40.54 μm. A greater degree of structural nonuniformity with 6x processing as opposed to 4x processing is indicated by the pattern of progressive material thinning on the X-axis as we proceed from Section 1 to Section 3. Section 1 exhibits a comparatively small Y-axis distance of 7.15 μm along the OY axis, suggesting moderate material thinning along the vertical axis; Section 2 (slice 2) shows an even smaller distance of 4.95 μm, suggesting even more discernible material thinning along this axis; and Section 3 displays a significant Y-axis material thickening of 15.76 μm, indicating a large variation in geometry, with a maximum material thickening along the vertical axis in this region. This shows a significant enlargement in part 3 after the initial material thinning on the Y-axis in Section 1 and Section 2, indicating a significant variation in geometry as a function of the surface position during the six-pass processing.

The following conclusions can be drawn from a comparison analysis of the two machining types, 4x_S and 6x_S, respectively: The 6x geometry shows progressive material thinning on the X-axis, particularly in Section 3, indicating that more passes make the geometry more compressed and variable in this direction; along the OY axis at 4x, Section 1 shows a large material thickening, but in Section 2 and Section 3, considerable material thinning is observed, suggesting a vertical non-uniformity in this geometry. Along the OX axis in the four-pass (4x) machining, the square geometry exhibits larger distances and a constant material thickening between sections, indicating a higher stability of the structure on the horizontal axis. There is noticeable material thinning in the first two parts when the number of passes on the Y-axis is increased to six. In section three, there is a significant broadening, indicating a considerable variability in the geometry along the vertical axis (depth). 

In summary, the four-pass processing results in a more extended and stable geometry on the OX axis, whereas only Section 1 exhibits a notable material thickening on the OY axis, with the remaining sections experiencing material thinning. In this instance, the geometry is more unstable since increasing the number of passes to six results in a greater variability on the OY axis and a more noticeable material thinning on the OX axis.

#### 3.6.3. Comparison of Hexagonal and Square Geometry at Four and Six Passes

In the case of the hexagonal geometry (4x and 6x) along the OX axis, the relative stability along the horizontal axis is suggested by the hexagonal geometry’s relatively constant distances along the X-axis, which range from 45.19 μm to 46.70 μm when machined with four passes. The distances on the X-axis change between 40.39 μm and 46.70 μm when the number of passes (6x) is increased, suggesting less material thinning but still a very stable structure.

In the case of the square geometry (4x and 6x) along the OX axis, when machined with four passes, the distances along the X-axis are considerably larger, ranging between 51.70 μm and 56.24 μm, suggesting a large stretching of the structure along this axis. When increasing the number of passes (6x), the X-axis distances decrease, ranging between 40.54 μm and 49.21 μm. This indicates a significant material thinning of the square structure after more passes.

The hexagonal shape is more stable in the X-axis with smaller deviations, indicating a high compressive strength in the horizontal direction, according to the result for the OX axis. The square geometry, however, is more sensitive to the quantity of passes. In contrast to four passes (4x), where the structure is substantially more stretched and uniform, a notable material thinning of the square structure along the X-axis is seen at six passes.

Four and six hexagonal geometries along the OY axis: The OY axis was found to have a significant material thinning in the vertical direction (depth) when machined by four passes (4x), with distances along the OY axis ranging from 2.66 μm to 7.78 μm. The hexagonal structure remained rather tight along the Y (depth) axis; however, the distances varied between 3.92 μm and 9.79 μm with a little less material thinning than at 4x when the number of passes was increased to 6x.

Square geometry (4x and 6x) along the OY axis: The distances along the Y-axis (depth) ranged from 12.45 μm to 20.87 μm when processed through four passes (4x), indicating a significant material thickening along the vertical axis. When the number of passes was raised to six, the distances ranged from 4.95 μm to 15.76 μm, indicating a considerable material thinning of the square geometry on the Y-axis.

The strong contrast in wettability between 4x_S and 6x_H arises from a transition between Cassie–Baxter and Wenzel wetting regimes driven by both the geometry and laser-induced chemistry. The 4x squared pattern has relatively shallow grooves and sharp edges that stabilize air entrapment beneath the droplet and keep the apparent contact angle high. In the 6x hexagonal pattern, the higher scan density deepens the valleys and rounds the rims while simultaneously increasing the surface energy through the mild oxidation/crosslinking of the polymer; these changes promote liquid penetration into the texture and a predominantly Wenzel state, which lowers the contact angle to about 30°. The selective increase in the COF in the six-pass samples is attributed to the stronger laser modification of the near-surface layer and more pronounced feature definitions. At this higher dose, the surface becomes harder and more brittle, local stress concentrations at asperity edges intensify, and the transfer film formed during sliding is more frequently disrupted, which elevates the average friction and its fluctuations. At the lower dose (4x), the deformation remains predominantly viscoelastic, debris is more effectively trapped by the texture, and the transfer film is more continuous, so the friction does not increase.

In conclusion, the hexagonal form at both velocities showed a noticeable material thinning for the OY (depth) axis, indicating a compact structure in this direction, regardless of the number of passes. At 4x, the square geometry was substantially more thickened on the OY axis than the hexagonal geometry; however, at 6x, it underwent significant material thinning, suggesting a greater sensitivity to a greater number of passes (6x) in the vertical (depth) direction.

## 4. Conclusions

Regardless of the texture type, the wettability test revealed that Arboblend V2 Nature had the same hydrophilic surface character (contact angle less than 90°). 

The base material’s wear behavior changed as a result of the LST surface modification. Only six passes with both textures resulted in higher COF values for all samples in the Arboblend V2 Nature example.

All samples showed an increase in weight following the degradation test. In general, if the polymer materials’ weight increases during a degradation test, it may be a sign of incomplete breakdown; if the degradation process is not finished, residues from partially broken-down polymer molecules may still be present, adding to the weight increase. Nevertheless, no degradation products were discernible from the microscopic examination of every sample following the degradation test.

At six passes, when the material thinning is more noticeable in the Y-axis (higher depths), the topographical side exhibits a significant degree of variability in the square geometry and a stable X-axis material thickening in the hexagonal geometry. In terms of geometry, the hexagonal shape is more stable and consistent in the material thickening on both axes, especially on the X-axis, where variations are less obvious. The hexagonal geometry has a better depth on the Y-axis but is less variable between sections than the square geometry, which is more thickened on the X-axis with both types of passes and experiences greater variations between sections, especially on the Y-axis, where the material thickening may be maximum in some sections and thinned in others. The square geometry is more sensitive to the number of passes, with significant variations at six passes and observable material thinning in some places.

## Figures and Tables

**Table 1:** LST process parameters.

Software	Cimita software
Laser	Diode-pumped solid state
Cut speed	1 [mm/s]
Cut passes	4 and 6 passes
Power	48 [mW]
Pulsation frequency	400 [Hz]
Wavelength	355 [nm]
Pulse width	6 [ps]

**Table 2:** Tests carried out on laser surface texturing.

No	Test Name	Equipment/Parameters/Investigations
1	Microscopic observation	Leica DVM6 digital microscope (DM)/ geometric structure of the surfaces, wear tracks.
2	Wettability test	Biolin Scientific Attension Theta Flex strain gauge/2 µL drops of distilled water measurement liquid were applied; three contact angle measurements as a function of time (60 s) were conducted; measurements were taken at 25 °C/contact angle (Δ) and surface wettability.
3	Wear test	CSM tribometer (CSM Instruments, Needham, MA, USA)/ball-on-plate method was used; a 6 mm-diameter Al_2_O_3_ ceramic ball was used; 4 N typical loads; sliding speed 1.2 cm/s, frequency 1 Hz, constant length of 6 mm/friction coefficient.
4	DSC analysis	The NETZSCH calorimeter type DSC 200 F3 Maia, with a temperature accuracy of 0.1 K, 276 sensitivities below 1 μW, and an enthalpy accuracy below 1% was used. Calibration was performed with Hg, Bi, In, Sn, and Zn according to standards. Heating was performed between RT and 180 °C for all samples using a heating rate of 10 °C/min. Sample fragments weighing less than 30 mg were cut and investigated under an Ar-protective atmosphere. The resulting DSC thermographs, comprising heat flow variations with temperature, were evaluated using the Proteus software v.6.1.
5	Degradation test	WKL 100/40—Weiss climate chamber/T↓–T = −5 °C wt = 1 h, aging: T = −5 °C, t = 3 h, T↑–T = 50 °C. HD = 90%. t =1 h, aging T = 50 °C. HD = 90%. t = 3 h/degradation.
6	Topography	Zegage-type profilometer with non-contact optical measurement technology/10× magnification; 0.815 µm lateral resolution and a 0.83/0.83 mm field of view (FOV).

**Table 3:** Contact angle for Arboblend V2 Nature.

Arboblend V2 Nature		Arboblend V2 Nature 4 Texturing Passes				Arboblend V2 Nature 6 Texturing Passes			
No of Samples	Contact Angle (°)	Texturing Type	No of Samples	Contact Angle (°)		Texturing Type	No of Samples	Contact Angle (°)	
				Average				Average	
1	63	Hexagon	1	65	62 ± 2	Hexagon	1	30	30 ± 8
2	61		2	62			2	40	
3	64		3	60			3	20	
Average	63 ± 1	Square	1	103	98 ± 4	Square	1	73	68 ± 5
			2	92			2	61	
			3	98			3	70	

**Table 4:** Summary of critical temperatures and absorbed heats for the melting process according to the DSC chart from Figure 7.

Sample	T_s gt_	T_mid gt_	T_i gt_	T_f gt_	ΔCp	T_s_	T_50_	T_f_	ΔH/m
	[°C]	[°C]	[°C]	[°C]	[J/(g·K)]	[°C]	[°C]	[°C]	[kJ/kg]
Base material	64	66.6	65.8	67.8	0.397	156.2	169.8	1761	−43.39
4xH	60.6	64.1	62.8	65.9	0.147	165.4	172.4	180.4	−40.43
4xS	62.1	65.4	64.5	67.3	0.241	169.7	174.2	180.3	−46.69
6xH	60.6	64.5	62.8	66.7	0.207	164.5	173.6	182.4	−44.51
6xS	62.4	66.3	64.6	67.7	0.141	168.3	175.1	181.9	−44.15

**Table 5:** Results of the degradation test.

Material		Weight (g)		Difference
		Before Texturing	After Texturing	
Arboblend V2 Nature	4 texturing passes	38.785	38.845	+0.06
	6 texturing passes	37.840	37.903	+0.063

**Table 6:** Statistics for hexagonal texture: 4x.

No.crt.	Reference Line	PV (µm)	RMS (µm)
1	Slice 1	52.93	5.13
2	Slice 2	16.39	1.81
3	Slice 3	28.45	1.96
4	Mean	32.59	2.97

**Table 7:** Statistical data: 6x_H, by geometric shape.

Nr.crt.	Reference Line	PV (µm)	RMS (µm)
1	Slice 1	21.75	2.281
2	Slice 2	13.41	1.553
3	Slice 3	34.36	2.776
4	Mean	23.17	2.203
5	Standard deviation	10.54	0.615
6	Range	20.94	1.223
7	3 Sigma	31.64	1.846

**Table 8:** Statistical data: 4x_S.

Nr.crt.	Reference Line	PV (µm)	RMS (µm)
1	Slice 1	25.27	3.818
2	Slice 2	24.95	4.124
3	Slice 3	28.23	3.630
4	Mean	26.15	3.857
5	Standard deviation	1.80	0.249
6	Range	3.27	0.494
7	3 Sigma	5.42	0.748

**Table 9:** Statistical data: 6x_S.

Nr.crt.	Reference Line	PV (µm)	RMS (µm)
1	Slice 1	26.56	2.812
2	Slice 2	16.59	1.463
3	Slice 3	22.27	2.326
4	Mean	21.81	2.200
5	Standard deviation	5.00	0.683
6	Range	9.97	1.349
7	3 Sigma	15.01	2.051

## Data Availability

The raw data supporting the conclusions of this article will be made available by the authors on request.
